# 

**Published:** 1857-05

**Authors:** 


*To Subscribers.*—All those indebted to us will receive with this number a bill for the amount due. The total indebtedness of our non-paying subscribers is now much larger than at any previous time, and very much larger than we can afford to carry along. This large sum is scattered over twenty states of the Union in small bills. We trust that each subscriber will make this a debt of honor, and remit immediately. We have formed the resolution not to encumber our editorial pages with repeated duns, and at some personal sacrifice have thus far lived up to the rule. Once a year we ask for what is our due—any further application will be made by means of circulars.

We may add, for the information of our western subscribers, or that large class of them who have been in the habit of paying to Mr. Jesse Clement, that that gentleman is no longer our agent, having removed to Dubuque, Iowa, to take charge of the *Dubuque Mercury*. The loss of the services of Mr. Clement will be a serious one to us unless each subscriber shall be his own agent; but we cannot part with one who has so long and faithfully served the Journal, without expressing the wish that he may find a pleasant home and warm friends in his new location.

